# How can the training of French midwives be revolutionized?

**DOI:** 10.18332/ejm/209554

**Published:** 2025-09-08

**Authors:** Lionel Di Marco

**Affiliations:** 1 Midwifery Department, Faculty of Medicine, Grenoble-Alpes University, Grenoble, France; 2 AGEIS Lab, EA 7407, Grenoble Alpes University, Grenoble, France; 3 National Conference of Midwifery Lecturers (CNEMa), Rouen, France

**Keywords:** training, midwives, university integration

Midwifery, in France, has a rich history that goes back several centuries and bears witness to the evolution of medical and social practices around childbirth. The French Revolution marked a turning point with the creation of the first official midwifery schools in 1802, at the instigation of Napoleon Bonaparte. The law of 1892^1^ which regulated the medical professions, gave midwives official status, although they were relegated to a non-medical classification until the twentieth century.

Until 2004, the core profession of French midwives was the physiology of pregnancy, childbirth, and the post-partum period. From that date, the skills of these professionals increased steadily, with a new law being passed every 2 to 3 years:

- Prescribing contraception^2^
- Role in the prevention of psychosocial risks (‘early prenatal interview’)
- Providing preventive gynecological check-ups for women in good health and taking part in breast and cervical cancer screening campaigns^3^
- Prescribing contraceptives
- Prescribing, and vaccinating women and then their children’s entourage and the children themselves
- Carrying out voluntary medical abortions
- Management of birthing centers
- Prescribing sick leave and treating sexually transmitted infections in women and their partners
- Performing instrumental abortions^4^

These successive laws reinforced the paradox of a profession that is being given more and more responsibilities and skills for promoting women’s and newborns’ health, but whose recognition in terms of salary, status, and scientific knowledge stagnates^5^.

During these 20 years of skill additions, and despite a reform of the training program in 2011–2013 incorporating a Master’s degree, the duration of studies remained unchanged, resulting in overloaded studies and a deterioration of student health. Figure 1 shows a timeline of French midwives’ skills.

**Figure 1:**
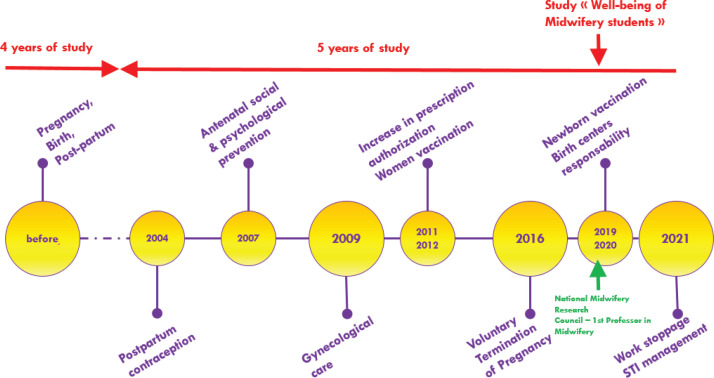
Brief history of midwifery skills. The timeline shows every addition of skills, without related modification of study duration, and despite the creation of academic jobs for midwives

After an alert of the National Association of Midwifery Students (ANESF in French), a report by the General Inspectorate of Higher Education and Research (IGESR)^6^ following a strike by French midwives, the national assembly approves a draft law to bring significant changes to the profession. The law #2023-29 of 25 January 2023^7^ provides for:

- The creation of a short third cycle of studies in midwifery sciences, which will be introduced for students entering the second year of the first cycle at the start of the academic year 2024 (Article 3)
- The creation of a State diploma for ‘doctors in midwifery’, awarded after validation of this third cycle and the successful defense of a thesis (Article 1)
- A revision of the training standards for the first and second cycles of studies in midwifery sciences (Article 3)

When this law was promulgated, the National Conference of Midwifery Lecturers (CNEMa in French) decided to implement an innovative strategy to meet the challenges of a profession undergoing profound changes. Indeed, a law remains ineffective until a decree and implementing orders are published: this was an opportunity for the profession to take control of its future.

## How to lead a French Revolution?

After analysis of the situation, the CNEMa concluded to maintain several guidelines throughout the management of the project.

This law had to be more than simply a re-engineering of training. It was supposed to make the profession more attractive and give greater consideration to research in midwifery, which is still in its infancy in France (in September 2024, there were only 16 academic teaching posts for around 25000 midwives, of whom only a few hundred had a PhD). Finally, this 6th year of training should be a springboard for genuine university integration, as less than a third of the 33 French structures are currently integrated into universities (the others are managed in hospitals).

In February 2023, the newly elected CNEMa Board decided to implement a bold strategy to achieve these far-reaching goals. Its aim was to:

- Follow the recommendations of the IGESR report concerning the introduction of a new training reference framework with a skills-based approach
- Initiate professional cohesion by including most of the French professional organizations in the discussions
- Focus our thinking on six areas: student life and well-being; re-engineering of studies; status of teachers; university integration; research; and status of clinical supervisors

In this way, the CNEMa has set up a working structure based on various collaborative tools (word processing files and spreadsheets; video-conferencing software; creation and management of mailing lists) and advanced planning of working meetings, modelled on the requests of the ministries responsible for drafting training decrees and orders.

By April 2023, 6 working groups had been set up with over 60 representatives from the main national professional organizations:

- CNEMa, coordinating all six groups
- ANESF
- French National College of Midwives (CNSF)
- National University Council - Midwifery Section (CNU-90)
- National Council of the Order of Midwives (CNOSF)
- National Union of Midwives (ONSSF)
- National Professional Council for Midwifery (CNP-Ma)
- National Association of Private Midwives (ANSFL)
- National Association of Coordinating Midwives (ANSFC)

French midwives had already taken the project into their own hands several months before the first Ministerial summonses.

## A minor ‘start-up dystocia’

In September 2024, the Ministry of Health and the Ministry of Higher Education and Research convened the first working group to draft the implementing regulations for the 2023-29 law. Unfortunately, the conditions proposed by the Ministries do not suit the midwifery organizations sitting on this working group. The Ministries wanted medical doctors to sit on the groups. The midwives were opposed to this, wishing to emancipate themselves: the first teacher-researchers in midwifery existed since 2020, and medical doctors have no longer been part of midwifery school boards since 2014.

The Ministries finally listened to these arguments, and after a delay (used to make progress on the various groups), the monthly meetings began in Paris. Between each meeting, validation and discussions were held with the working groups coordinated by the CNEMa. Very quickly, several limits were placed on the discussions, since the ministries wished to work exclusively on the reengineering of training. However, a holistic view of the situation seemed necessary, since the reengineering of training courses would require parallel discussions on the status of training supervisors, transport allowances, and the operation of training structures.

## A need for ‘labor management’

Fortunately, the working groups had completed a large part of the re-engineering process: an initial proposal for a training reference framework for the three training cycles already existed in December 2024. The first stage in the discussions was to convince everyone that the skills-based approach was the best option. Indeed, the IGAS/IGESR report proposed this option, but several concerns were initially raised:

- Could a long training course use this approach?
- Did the 33 French midwifery schools have the resources to be able to change their training standards
- so radically?
- Was this approach going to lead to a real standardization of training across the country?

Other countries (India, Canada) have already introduced this type of approach in medical training courses^8-11^. Since 2023, the CNEMa has represented 100% of midwifery schools in France and will be able to facilitate the implementation of this ambitious re-engineering program. This meant that midwifery training would become the first (and only) long course in French healthcare to offer a skills-based curriculum. Six major areas of competence have been identified:

- Antenatal monitoring: ensuring medical follow-up of the pregnancy
- Perinatal care: providing medical care during childbirth and after birth
- Postnatal care: providing medical care during the postnatal period
- Gynecological monitoring: providing gynecological monitoring for prevention and contraception, and promoting sexual and reproductive health
- Research: applying a scientific approach to medical practice
- Generic skills: acting as a responsible public health and medical professional

## Hard ‘stagnation of dilatation’

The provisional text takes a long time to take shape, in an incessant back-and-forth ballet. It was necessary to find wording that respects the freedom of the universities^11^. While at the same time imposing a national standard for midwifery training. As a result, certain formulations are not in line with midwifery practice. For example, in French, the verb *accoucher* means ‘to give birth’ as much as ‘to deliver babies’, which has led to several discussions between birth professionals and non-professionals. Similarly, a lack of midwifery knowledge led to misunderstandings, such as limiting birth assistance to the simple management of pain using epidural analgesia.

In addition, student working time during internships was a source of misunderstanding and stumbling blocks. Midwives wanted flexibility so that students could follow the schedules of their training supervisors, who sometimes work 12-hour shifts 3 or 4 times a week. After several discussions on the subject, educational coherence took precedence. In addition, national uniformity would be guaranteed by a set number of hours of internships per cycle.

## An inspiring experience for midwives around the world?

In the end, the most ambitious reform of French midwifery training in history saw its implementing legislation published more than 17 months after its enactment, a delay that far exceeds the duration of a pregnancy.

At a time of historic opportunity to revise the EU Directive that regulates the minimum standards for midwifery^12^, a text published on 3 July 2024^13^ shows how French midwives skills will be validated during initial studies. Skills are broken down into the six areas presented in this article, and are expressed in terms of professional situations, with a progressive development pathway over the three training cycles. Table 1 shows, in an example, the breakdown of the skills to be validated cycle by cycle.

**Table 1:** Midwifery skills development trajectory

*Area of expertise: Antenatal care*	
**Skill 1: Monitoring and managing low-risk pregnancies by carrying out various antenatal monitoring consultations and obstetric emergency consultations**	
	*Cycle 1: Identify the different stages of antenatal and emergency obstetric care, being able to*: Welcome the woman and/or the couple and explain the consultation procedure Carrying out an interview and clinical examination Analyzing clinical and paraclinical data to provide advice or information tailored to the situation and identify the additional tests and treatments required
	*Cycle 2: Carry out the various antenatal follow-up consultations and obstetric emergencies in everyday situations:* Screening for risk factors and vulnerabilities that have an impact on pregnancy Diagnose benign pregnancy-related disorders .../... Evaluate and adapt the strategy in place
	*Cycle 3: Carrying out various antenatal follow-up consultations and obstetric emergencies in complex situations:* Drawing up an assessment of the situation of the pregnant woman and the fetus, based on all the clinical and paraclinical data, to prescribe the additional tests and therapies required and refer the patient to the appropriate professionals and structures .../...
**Skill 2: Assists women and couples with their childbirth plans by conducting early prenatal interviews and designing and running birth and parenthood preparation sessions**	
	*Cycle 1*: *Participating in early prenatal interviews and leading birth and parenthood preparation sessions:* Identify the support needs of the pregnant woman/couple Screening and providing information on risk behavior Co-leading workshops on perinatal practices
	*Cycle 2: .../...*
	*Cycle 3: .../...*

Elements in italics are given as examples and do not form part of the text published in French law (for details of the text of the law, please refer to reference 13).

The #2023-29 French law calls for all these points to be implemented by September 2027. In fact, over and above the simple addition of a 6th year of study and the creation of a ‘Practicing Doctorate in Midwifery’ (This *doctorat d’exercice* is specific to France and does not correspond to a PhD, nor does it give the prerogatives of a PhD), the aim is to:

- Integrate all 33 training structures into universities
- Consider the careers of teachers and develop research in midwifery
- Improve the attractiveness of a profession that is essential to the health of women and the population, and whose skills have been increasing for 15 years without acknowledgement

This text specifies the totality of the training during the first cycle: 2 cycles therefore remain to be formalized, which French midwives hope to bring into the world soon.

## Data Availability

Data sharing is not applicable to this article as no new data were created.
